# Analysis of Antiemetic Use After Initiation of Hormone Therapy

**DOI:** 10.1001/jamanetworkopen.2022.11883

**Published:** 2022-05-12

**Authors:** Martin Torp Rahbek, Erika Björkström Gram, Jesper Hallas, Mette Marie Hougaard Christensen, Lars Christian Lund

**Affiliations:** 1 Department of Clinical Pharmacology, Odense University Hospital, Odense, Denmark; 2 Clinical Pharmacology, Pharmacy and Environmental Medicine, Department of Public Health, University of Southern Denmark, Odense, Denmark.

## Abstract

This cross-sectional study investigates the occurrence of the prescribing cascade of antiemetic after hormone therapy.

## Introduction

A prescribing cascade describes when a drug is associated with an adverse event, which is subsequently treated with an additional drug, leading to polypharmacy and other potential harms. For example, hormone therapy (HT), commonly used for menopausal symptoms, can be associated with nausea and, in turn, prescription of an antiemetic, such as metoclopramide. We aimed to quantify the occurrence of this prescribing cascade.

## Methods

This cohort study using sequence symmetry analysis (SSA) was approved by the University of Southern Denmark institutional data-protection board and the Danish Health Data Authority. By Danish law, pure register studies do not require ethics committee approval or informed consent. This study was reported according to the STROBE reporting guideline.

Using administrative data from the Danish National Prescription Registry,^2^ we performed an SSA^3^ evaluating whether women initiating HT were at an increased risk of subsequently initiating antiemetics. The SSA compared the sequence of the initiation of an exposure (ie, HT) and outcome drug (ie, those used to treat nausea, including sedating antihistamines, propulsives, or antiemetics, hereafter all referred to as *antiemetics*) (eTable in the Supplement). If there was no cascade, the sequence of HT then antiemetics would be just as frequent as the reverse. If there was a prescribing cascade, the sequence HT then antiemetics would be more frequently observed. The symmetry principle was previously used in prescribing cascade analyses.^4^

We identified all women in Denmark aged 45 to 59 years who redeemed a first prescription of oral, transdermal, or vaginal HT (ie, estrogens) from 1997 to 2017 and initiated an antiemetic 180 days before or after HT (eTable in the Supplement). Using logistic regression, we estimated sequence ratios with 95% CIs overall and stratified on route of HT administration and type of antiemetic. Vaginal HT was used as a negative control given that systemic absorption is negligible.^5^

Risk of prescribing cascade was estimated as the difference between 2 possible sequences divided by total number of individuals who initiated HT. Data were analyzed on December 21, 2021.

## Results

We identified 129 586 women who initiated systemic HT, of whom, 2042 women (median [IQR] age, 50 [48-53] years) started taking an antiemetic within 6 months before or 6 months after HT. Most women received oral HT, with 250 women (12.24%) initiating transdermal therapy. The most common outcome drug was metoclopramide, involved in 1630 sequences (79.82%). Overall, 1104 women initiated HT before antiemetics and 938 women had the reverse sequence, yielding a sequence ratio (SR) of 1.18 (95% CI, 1.08-1.28). Prescribing cascade occurred in 132 of 105 361 oral route sequences (risk, 0.13%) and 34 of 14 668 transdermal route sequences (risk, 0.23%) using any antiemetic as outcome. The SR was 1.31 (95% CI, 1.02-1.69; 142 HT first/108 HT last sequences) for transdermal HT and 1.16 (95% CI, 1.06-1.27; 962 HT first/830 HT last sequences) for oral HT. We found increased SRs when considering only metoclopramide as the outcome drug for transdermal HT (1.54 [95% CI, 1.15-2.06]; 114 HT first/74 HT last sequences) and oral HT (1.16 [95 % CI, 1.05-1.29]; 775 HT first/667 HT last sequences). We found no association between vaginal HT and antiemetics (SR, 0.96 [95% CI, 0.88-1.06]; 807 HT first/837 HT last sequences) (Table). Oral and transdermal administration exhibited a temporal asymmetry on visual inspection, while vaginal administration did not (Figure).

**Table.** Sequences and SRs of HT and Antiemetic Therapy by HT Administration Route

Route of HT administration	Sequence of HT and antiemetic therapy, HT first/HT last	SR (95% CI)
**Any antiemetic therapy**		
Oral or transdermal (systemic)		
Total	1104/938	1.18 (1.08-1.28)
Oral	962/830	1.16 (1.06-1.27)
Transdermal	142/108	1.31 (1.02-1.69)
Vaginal	807/837	0.96 (0.88-1.06)
**Metoclopramide therapy**		
Oral or transdermal (systemic)		
Total	889/741	1.20 (1.09-1.32)
Oral	775/667	1.16 (1.05-1.29)
Transdermal	114/74	1.54 (1.15-2.06)
Vaginal	600/593	1.01 (0.90-1.13)

Abbreviations: HT, hormone therapy; SR, sequence ratio.

**Figure.**
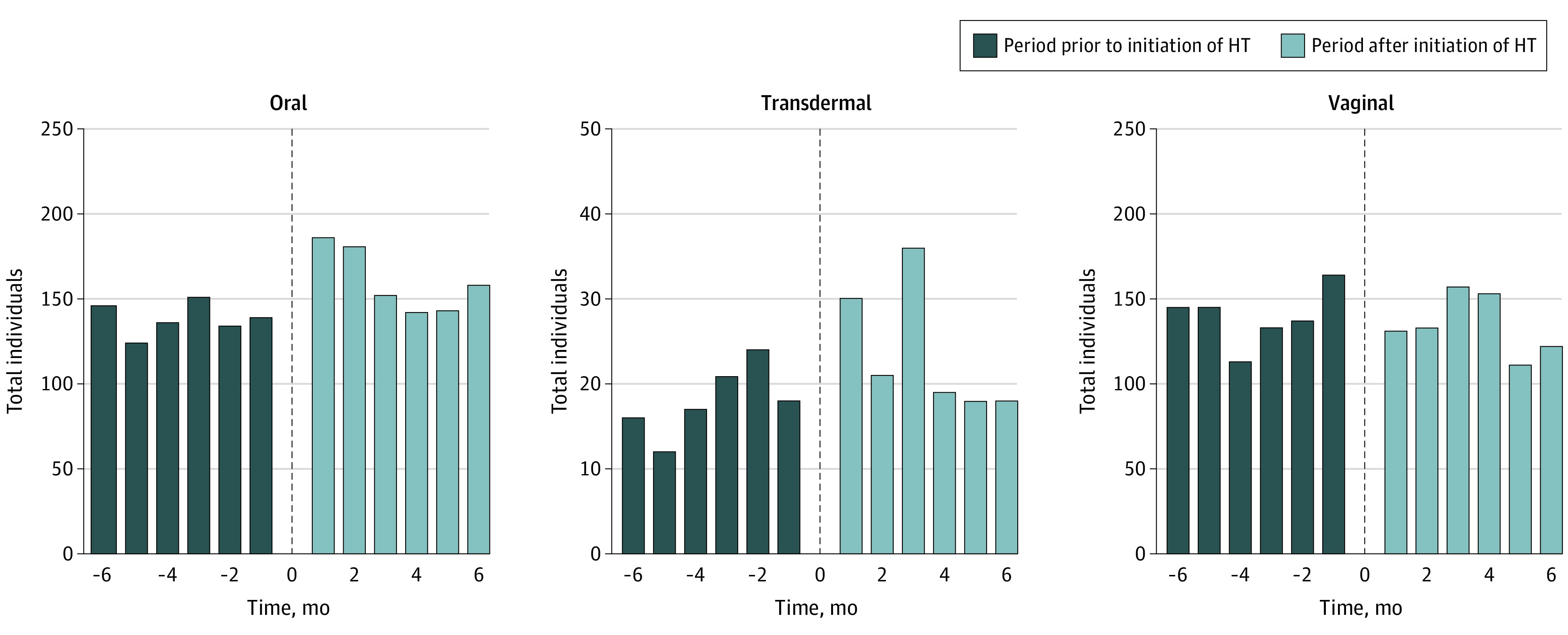
Individuals Initiating Antiemetics by Hormone Therapy (HT) Administration Route X-axis indicates time before and after initiation of HT; zero on the x-axis, initiation of HT.

## Discussion

In this cohort study using SSA, we identified a prescribing cascade between initiation of HT and antiemetics. Our results align with prior evidence on adverse events^1^ but add to current knowledge by quantifying risk of prescribing cascades and finding increased risk with transdermal HT vs oral and vaginal HT. Oral HT undergoes first-pass metabolism, while transdermal HT does not. Depending on individual variation in dermal absorption,^6^ this may be associated with increased variability in peak concentration of HT, which may be associated with increased risk of nausea and hence antiemetic therapy. A limitation of our study is its nonrandomized nature and inability to identify patients who developed nausea but did not initiate antiemetic treatment.

Our results suggest that HT-related nausea may be treated with antiemetics instead of dose reduction or discontinuation. This prescribing cascade is an unintended outcome associated with HT, which our results suggest may be avoided.

## Notes

Supplement. **eTable.** Anatomical Therapeutic Chemical Codes Used to Define Exposure and Outcomes in Danish National Prescription Registry
