# Error in Author Name in Byline and Supplement

**DOI:** 10.1001/jamanetworkopen.2025.40719

**Published:** 2025-10-07

In the Research Letter titled “Analysis of Antiemetic Use After Initiation of Hormone Therapy,”^1^ published May 12, 2022, the name of author Erika Björkström Gram appeared incorrectly as Erika Angelica Björkström Gram in the article byline and as Gram EAB instead of Gram EB in the Supplement. The article and supplement have been corrected.^1^

## References

[1] Rahbek MT, Gram EB, Hallas J, Christensen MMH, Lund LC. Analysis of antiemetic use after initiation of hormone therapy. JAMA Netw Open. 2022;5(5):e2211883. doi:10.1001/jamanetworkopen.2022.11883 35552727 PMC9099429

