# Supplementary material for: Analysis of Antiemetic Use After Initiation of Hormone Therapy
Source: JAMA Netw Open. 2022 May 12;5(5):e2211883. doi: 10.1001/jamanetworkopen.2022.11883 (PMC9099429; doi:10.1001/jamanetworkopen.2022.11883)
Supplement: Supplement. — eTable. Anatomical Therapeutic Chemical Codes Used to Define Exposure and Outcomes in Danish National Prescription Registry [file jamanetwopen-e2211883-s001.pdf]

Supplemental Online Content

Rahbek MT, Gram EB, Hallas J, Christensen MMH, Lund LC. Analysis of antiemetic use after initiation of hormone therapy. *JAMA Netw Open.* 2022;5(5):e2211883. doi:10.1001/jamanetworkopen.2022.11883

eTable. Anatomical Therapeutic Chemical Codes Used to Define Exposure and Outcomes in Danish National Prescription Registry

This supplemental material has been provided by the authors to give readers additional information about their work.

© 2022 Rahbek MT et al. *JAMA Network Open.*

eTable. Anatomical Therapeutic Chemical Codes Used to Define Exposure and Outcomes in Danish National Prescription Registry

Variable	Codes
Hormonal therapy	G03CA, G03F
Drugs used to treat nausea	
Sedating antihistamines	R06AE03 (Cyclizine), R06AE05(Meclozine), R06AD02(Promethazine)
Propulsives	A03FA01(Metoclopramide), A03FA03(Domperidone)
Antiemetics	A04AD01(Scopolamine), A04AA01(Ondansetron)

© 2022 Rahbek MT et al. *JAMA Network Open.*
